# Diazotization-Coupling Reaction-Based Determination of Tyrosine in Urine Using Ag Nanocubes by Surface-Enhanced Raman Spectroscopy

**DOI:** 10.3390/nano8060400

**Published:** 2018-06-03

**Authors:** Yudong Lu, Dechan Lu, Ruiyun You, Jialing Liu, Luqiang Huang, Jingqian Su, Shangyuan Feng

**Affiliations:** 1 Fujian Key Laboratory of Polymer Materials, College of Chemistry and Materials Science, Fuzhou 350007, Fujian, China; 18149544204@163.com (D.L.); youruiyun@fjnu.edu.cn (R.Y.); 2 Center of Engineering Technology Research for Microalgae Germplasm Improvement of Fujian, Southern Institute of Oceanography, Fujian Normal University, Fuzhou 350117, Fujian, China; 18850383089@163.com (J.L.); sjq027@fjnu.edu.cn (J.S.); 3 Fujian Key Laboratory of Innate Immune Biology, Biomedical Research Center of South China, College of Life Science, Fujian Normal University, Fuzhou 350117, Fujian, China; 4 Key Laboratory of Optoelectronic Science and Technology for Medicine of Ministry of Education, Fujian Provincial Key Laboratory of Photonics Technology, Fujian Normal University, Fuzhou 350117, Fujian, China; syfeng@fjnu.edu.cn (S.F.)

**Keywords:** silver nanocubes, diazotization-coupling reaction, Tyrosine, Surface enhanced Raman scattering

## Abstract

A novel, simple, and highly sensitive method was developed to detect the concentration of tyrosine-derived azo dye indirectly using silver nanocubes (AgNCs) as a substrate on a super-hydrophobic silver film by surface-enhanced Raman spectroscopy (SERS). Diazotization-coupling reaction occurred between diazonium ions and the phenolic tyrosine, resulting in three new typical peaks in the SERS spectrum of the azo dye that was formed on the AgNCs, indicating strong SERS activity. Subsequently, the limit of detection of this approach was as low as 10^−12^ M for tyrosine. Moreover, the SERS intensities of the three typical SERS signals of the analyte were linearly correlated with the logarithm of concentration of the Tyrosine. The proposed method shows great potential for tyrosine detection in the urine samples of normal humans.

## 1. Introduction

Tyrosine (Tyr) is one of the aromatic acids in the human body, and it is essential for the successful synthesis of neurotransmitter serotonin [1]. A lack of Tyr may lead to albinism [2] and alkaptonuria [3]. In recent years, clinical trials in hospitals have shown that Tyr levels in the urine of colorectal cancer patients, including adenoma cancer patients, are significantly higher than in that in the urine of normal humans. When compared with the traditional tumor marker carcino-embryonic antigen (CEA), Tyr detection in urine has higher sensitivity in colorectal cancer screening. Accordingly, many testing methods have been used, such as liquid chromatograph-mass spectrometer ( LC-MS)/MS, which was optimized for human urine with a lower limit of detection (LOD) of 1 nmol/L [4]; Ultra performance liquid chromatography-fluorescence detector( UPLC-FLD), a rapid and practical method for simultaneous determination of Tyr in human urine with a LOD of 0.016 mg/L [5]; and FI-direct chemiluminescence proposed for the detection of Tyr, a new procedure that has good sensitivity with an LOD of 50 ppb [6]. However, these methods require a long time, a sufficient amount of the sample, and complicated procedures for sample pretreatment.

Surface-enhanced Raman scattering (SERS) is particularly attractive as a practical analytical tool, owing to its high sensitivity, strong selectivity, and rapid diagnosis of diseases. SERS has been widely used in many fields, including in vitro diagnosis of cancer [7], non-invasive detection of colorectal cancers and nasopharyngenal cancers [8,9], detection of aromatic pollutants in water [10], and monitoring of intracellular melanogenesis [11]. As metal nanoparticles are used as SERS substrates [12], the metal nanostructure may be one of the factors that affect SERS signal enhancement, such as Au and Ag nanostars [13], nanoflowers [14], and nanocubes (NCs) [15]; these irregular microstructures can significantly form SERS hotspots. When compared with nanospheres, silver NCs (AgNCs) show superior SERS enhancement because they are characterized by regular juxtaposed facets: eight sharp corners and twelve edges [16]. The light field is located at the corner, which greatly enhances the strength of the electric field and generates the hot spot effect. AgNCs have been used to detect 2,4-dinitrotoluene (DNT) [17] and pesticides [18], and they have potential biosensing applications [19].

Because of the small scattering cross section and large polarity, the SERS signal of Tyr is weak. Tyr with low concentrations and non-labeling direct detection often does not result in an excellent SERS signal, even when relying on the substrate that has a large number of hot spots. The SERS labeling method has high sensitivity and selectivity, but the labeling process of probe molecules and specific molecules on the SERS substrate is complicated and time-consuming, which is not suitable for the detection of practical samples. One method is to derivatize Tyr and convert the target molecule into a chromophore with a large scattering cross section, and become a new substance that is easy to get SERS signals. Complex separations or marking of probes are not convenience in complex samples. To use the SERS technology for ultrasensitive detection of Tyr, Raman signals can be indirectly detected by some other methods, such as Tyr phosphorylation [20] or nitration of Tyr [21]. Cheng [22] have reported that using silver nanoparticle-treated filter paper as substrate for detection of Tyr, the limit was 6.25 × 10^−7^ M. However, this method has no specificity and is not suitable for use in complex samples. Here, we adopted the azo coupling reaction to detect Tyr, wherein a diazonium salt reacts with an electron-rich aromatic nucleus to produce an azo compound, which is known as an azo dye. There are several excellent reviews on surface-enhanced resonance Raman scattering (SERRS) assay for Tyr and histidine [23], phenolic estrogens [24], and thyrotropin-releasing hormone [25] based on azo coupling.

Further, a super-hydrophobic silver film is a powerful tool for reducing the coffee ring effect caused by the diffusion of analytes. Through the shrinkage of the super-hydrophobic silver film, diffused analytes can be enriched to detect trace molecules. Most SERS substrates do not have much hydrophobicity, and a drop of sample spreads over the large area on substrate. Laser Raman spectroscopy that is used for Raman detection has a very small laser spot area, resulting in most of the molecules still remaining undetected. As the solvent continues to evaporate, the volume of the liquid continues to decrease, and the molecules to be measured are concentrated and enriched in the SERS host spot. It has been reported that 10^−18^ M Rhodamine 6G (R6G) can be detected using a super hydrophobic substrate [26]. In addition, the adsorption of analytes and nanosilver colloids on super-hydrophobic silver films can be monitored in real time. Yang [27] developed dynamic SERS (D-SERS) for the detection of optimal hotspots; the best time at which the spectral intensity is stabilized can be detected by D-SERS. The interaction between nitrogen and nitrogen bonds in the diazo coupling reaction is weak at extremely low concentrations, which results in weak SERS enhancement; however, the detection limit can be increased with super-hydrophobic substrate enrichment.

In this work, AgNCs were combined with a super-hydrophobic silver film to detect Tyr based on the diazotization coupling reaction. To accomplish this reaction, Tyr coupling with diazotized *p*-amino thiophenol (PATP) under the action of Pauly’s reagents to form azo bonds, and the concentration of Tyr was determined directly from the azo bonds. The performance of AgNCs to determine the concentration of Tyr in human urine was evaluated.

## 2. Materials and Methods

### 2.1. Materials and Instrumentation

Silver nitrate (AgNO_3_) was purchased from Sinopharm Co., Ltd. (Guangzhou, China). Tyrosine was purchased from Shenggong Biological Engineering Co., Ltd. (Shanghai, China). PATP, cetyltrimethyl ammonium bromide (CTAB), Rhodamine 6G (R6G), and sodium carbonate (Na_2_CO_3_) were purchased from Aladdin Co., Ltd. (Shanghai, China). Glucose and aqueous ammonia were purchased from Xilong Scientific Co., Ltd. (Guangzhou, China). Sodium nitrite (NaNO_2_) was purchased from Fuchen Chemical Reagents Factory (Tianjin, China), and hydrochloric acid (HCl) was purchased from Xuri Chemical Company (Zhejiang, China). SERS spectra were obtained using (Renishaw, Gloucestershire, UK) confocal Raman spectrometer with 785-nm laser excitation source. Transmission electron microscopy (TEM) images of AgNCs were obtained using a JEOL JEM-2100 (Tokyo, Japan), the size of the super-hydrophobic silver film was by emission scanning electron microscopy JSM-7500F (Tokyo, Japan). UV-vis absorption spectra were recorded with a UV-LAMBDA 950 (PerkinElmer, Shelton, CT, USA) spectrophotometer.

### 2.2. Preparation of AgNCs Substrates

AgNCs were synthesized by a one-step hydrothermal method [16]. Briefly, [Ag(NH_3_)_2_]OH was prepared by adding dilute ammonia to an aqueous solution of AgNO_3_ (0.01 M), and the solution turned clear from turbid. Then, 5 mL CTAB (20 mM) and 10 mL glucose (10 mM) were rapidly added into 5 mL of [Ag(NH_3_)_2_]OH solution and magnetically stirred for 5 min. The mixed solution was then transferred to a 25-mL autoclave and heated at 180 °C for 8 h. Finally, the autoclave was taken out from the drying oven and was cooled naturally to room temperature. The nanoparticles were separated from the solvent by centrifugation (10,000 rpm for 20 min) and washed with deionized water to remove excess CTAB. This process was repeated three times. According to reports that CTAB serves as a stabilizing agent to avoid particle aggregation and aggregation-induced SERS enhancement, and that CTAB is a nonaromatic surfactant with low Raman cross section and low spectral background [28]. CTAB plays a crucial role in the formation of AgNCs, but the SERS signal may be affected by excess CTAB.

### 2.3. SERS-based Diazotization-Coupling Reaction Test

The reagents that were used in the diazotization-coupling reaction were prepared, as previously reported [23]. Reagent A1 was prepared by dissolving *p*-amino thiophenol (10^−3^ M) in 0.5 mL HCl (12 M) to prepare a 50mL solution, reagent A2 was prepared by dissolving NaNO_2_ in H_2_O (5%, *w*/*v*), and reagent B was prepared by dissolving Na_2_CO_3_ in H_2_O (8%, *w*/*v*). All of these reagents were stored at 4 °C prior to use. For the diazotization-coupling reaction, the mixing ratio of reagent A1: reagent A2: reagent B: Tyr was 1:1:1:2 (*v*/*v*/*v*/*v*).

Firstly, reagent A2 was added to reagent A1 under magnetic stirring in an ice-water bath for 1 min. Then, a Tyr sample and reagent B were rapidly added to the above solution with stirring for another 1 min. After the completion of coupling reaction, 1.5 μL of the obtained mixture mixed with 1.5 μL of AgNCs was immediately deposited dropwise onto a super-hydrophobic silver film. Finally, the analyte was exposed to a laser power of 90 μW for SERS measurement.

### 2.4. SERS-Based Diazotization-Coupling Reaction and Principle

**Figure 1:**
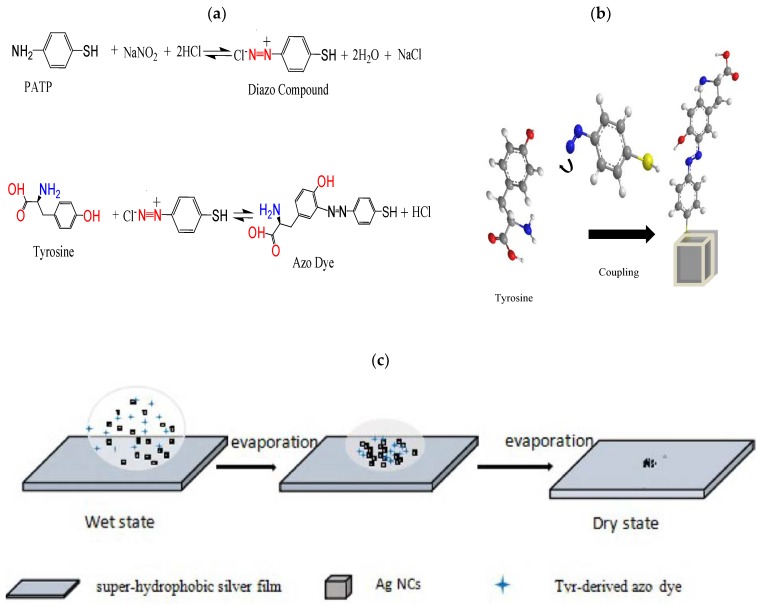
Schematic illustration of detection of Tyrosine (Tyr)-derived azo dye on a super-hydrophobic silver film coated with silver nanocubes (AgNCs) as a substrate. (**a**) Azo coupling reaction; (**b**) Azo coupling and Surface-enhanced Raman scattering (SERS)-based Tyr detection; (**c**) Dynamic contraction process of the analyte on the super-hydrophobic silver film.

The mechanism for ultrasensitive detection of Tyr based on azo coupling is shown in Figure 1a. PATP reacts with NaNO_2_ under acidic conditions and a low temperature to generate a diazo compound, which can easily produce azo dyes in alkaline environments. It should be noted that several aspects of this reaction require attention: (1) Reaction should be carried out at low temperatures to avoid decomposition, (2) reaction should be performed in strong acidic media to prevent diazo compound coupling, and (3) excess NaNO_2_ will promote diazonium salt decomposition. Moreover, since the reaction is unstable, the test time must be controlled.

In the sensitive SERS-based methodology, together with the azo coupling reaction for Tyr detection, the azo molecules are strongly adsorbed by –SH on the AgNCs, as shown in Figure 1b. Sui et al. [23] reported a rapid and ultrasensitive SERRS assay for histidine and Tyr based on azo coupling, in which synthesis and testing is completed within 2 min. They should be tested when the analytes are in wet state. According to our knowledge, the Raman enhancements in the wet state are not good. If it is possible to extend the detection time and to monitor the Raman intensities reach stability in situ real time, so that we can detect the target at the stable time. In recent years, our group has developed a super-hydrophobic silver film. Briefly, silver film was plated on a glass plate by a silver mirror reaction and then modified with dodecanethiol to obtain a super-hydrophobic silver film. As its sensitivity and reproducibility have been verified, it can be used as a platform for D-SERS for the dynamic contraction process, as shown in Figure 1c. First, the analyte was mixed with AgNCs on the super-hydrophobic silver film, which was transformed from the wet state to the dry state for SERS measurements.

## 3. Results and Discussion

### 3.1. Characterization of the Prepared AgNCs

**Figure 2:**
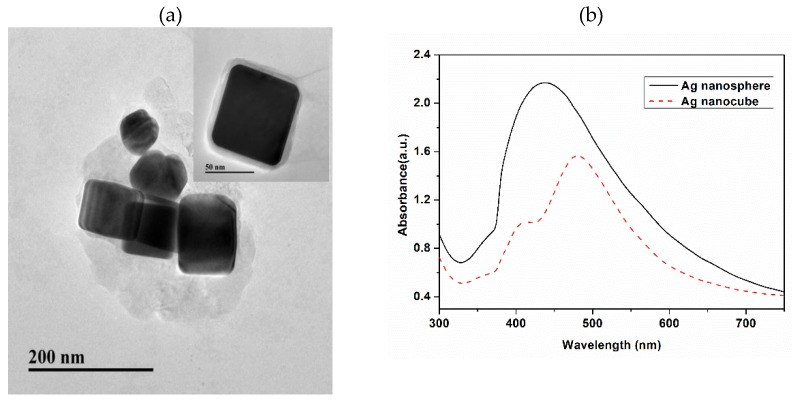
(**a**) AgNCs TEM image and (**b**) UV-Vis absorbance spectrum of AgNCs and nanosphere.

**Figure 3:**
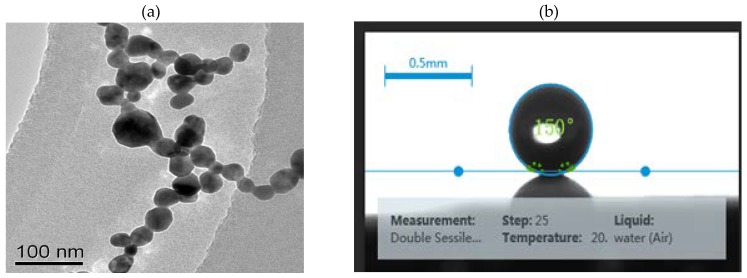
(**a**) Transmission electron microscopy (TEM) image of Ag nanospheres and (**b**) Static contact angle of the super-hydrophobic silver film.

The TEM images of the AgNCs are shown in Figure 2a. The AgNCs, synthesized using the one-step hydrothermal method, were 75 nm in diameter. AgNCs with a smooth surface, clear edges, and corners can be seen. Ag spheres were prepared by the deoxidizing method using hydroxylamine hydrochloride and silver nitrate, as reported by Leopold [29]. Figure 3a shows a transmission electron microscopy (TEM) photograph of the prepared silver nanospheres, the particle sizes with a mean diameter of 40 nm. Figure 3b shows that super-hydrophobic silver film with a static contact angle of 150°. From the average geometrical parameters of AgNCs, we calculated their average volume (4.21 × 10^5^ nm^3^). When considering 10 mM as the concentration of Ag, and 1.19 × 10^−13^ g as the mass of a single AgNCs, we obtain the number concentration of AgNCs as 8.07 × 10^9^/mL [30]. The UV-vis absorption spectra of AgNCs and Ag nanospheres are shown in Figure 2b. For the AgNCs, two notable absorption of 405 and 480 nm. The Ag nanospheres exhibit the characteristic maximum absorption wavelength at 430 nm.

**Figure 4:**
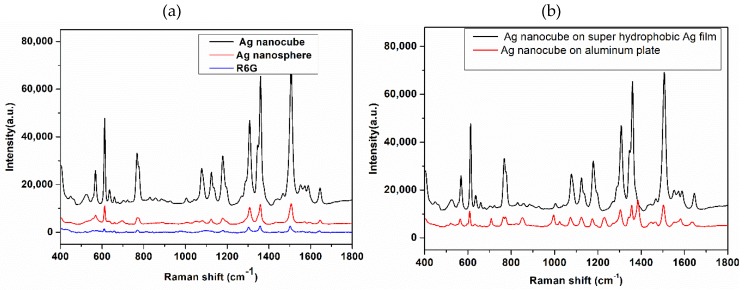
(**a**) SERS spectra of 10^−7^ M R6G mixed with AgNCs and Ag nanospheres on the super-hydrophobic silver film and the normal Raman spectra of R6G powder; (**b**) SERS spectra of 10^−7^ M R6G mixed with AgNCs on the super-hydrophobic silver film and the aluminum plate.

When compared with Ag nanospheres in Figure 4a, AgNCs have higher sensitivity. The Raman spectra of two kinds of silver NPs mixed with R6G (10^−7^ M) deposited on super-hydrophobic silver film, typical peaks of R6G can be clearly identified under the effect of AgNCs enhancement, and the SERS intensity of Ag nanospheres is lower than that of AgNCs. The enhancement factor is one of the most important parameters for the SERS effect. The Raman spectrum of R6G powder was detected to estimate the enhancement factor of silver NCs. It can be seen that the SERS effect of the AgNCs is conspicuous when compared to that of the R6G powder. The intensities of Raman peak at 1386 cm^−1^ for the SERS and normal Raman spectra were 51852 and 2139, the enhancement factor was 10^9^. The detailed calculation process can be found in previous work [17,31,32].

(1) $$\mathrm{EF}=\frac{I_{\mathrm{SERS}}\times N_{\mathrm{bulk}}}{I_{\mathrm{RS}}\times N_{\mathrm{SERS}}}$$

where *I*_SERS_ is the SERS intensity of the analyte (in this case R6G). *I*_Raman_ is the normal Raman intensity of the R6G measured over a aluminum plate. Whereas, *N*_SERS_ is the number of molecules that were probed in SERS. Whereas, *N*_bulk_ is the powder of the analyte sample.

*N*_SERS_ = *CVN*_A_*S*_scan_/*S*_sub_ (2)

*N*_bulk_ = *M*ρ*hN*_A_*A*_Raman_ (3)

*C* is the molar concentration for analyte solution, *V* is the volume of the droplet = 3 μL in SERS, *S*_scan_ is the area of Raman scanning, *S*_scan_ = 5.0 μm^2^, and *S*_sub_ is the area of the substrate, *S*_sub_ = 9 mm^2^. *A*_Raman_ is the laser spot diameter, was calculated for 785 nm using the formula (laser spot diameter = 1.22λ/NA), NA = 0.75, so *A*_Raman_ = 1.28 μm, *h* is the confocal depth, *h* = 2 μm. In addition, we compared the SERS enhancement effect of super-hydrophobic silver film under the same experimental conditions; AgNCs mixed with R6G (10^−7^ M) deposited dropwise onto an aluminum pan are illustrated in Figure 4b. We can clearly see that the super-hydrophobic silver film has an excellent SERS enhancement.

**Figure 5:**
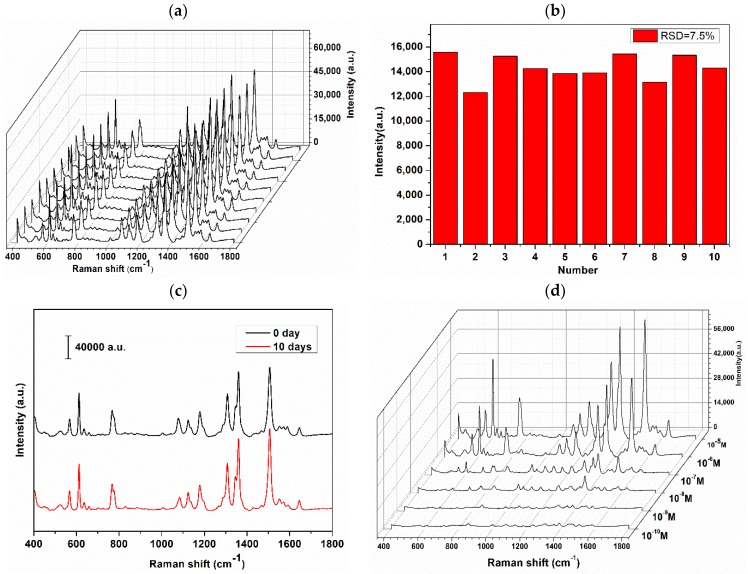
(**a**) The waterfall plot of SERS spectra (randomly selected areas) for R6G(10^−7^ M) obtained from 10 times measurement; (**b**) Relative standard deviation (RSD) of specific Raman modes at 1181 cm^−1^ of the 10 random points; (**c**) SERS signals of R6G (10^−7^ M) mixed with AgNCs on the super-hydrophobic silver film, substrates under the exposure to the common air environment for 0 day and 10 days; and, (**d**) SERS spectra of R6G was deposited from solutions with different concentrations (10^−5^ to 10^−10^ M) mixed with AgNCs on the aluminum plate.

To evaluate whether the AgNCs features excellent reproducibility under the low concentration of R6G (10^−7^ M) from randomly selected potions, R6G was mixed with AgNCs were deposited on super-hydrophobic silver film, as shown Figure 5a. The signal reproducibility of R6G is pretty good and standard deviation is only 7.5% in Figure 5b, which brings reliable SERS detection. Figure 5c illustrate the SERS signals from substrates under the exposure to the common air environment for 0 day and 10 days, we can clearly see that the Raman signals has not diminished. In consequence, we can conclude that AgNCs suffer from exposure to air, which could not result in a degradation of the particles. The efficiency of AgNCs was also evaluated using AgNCs modified with different concentrations of R6G drops on the aluminum plate. As the R6G concentration decreases, the Raman signal becomes weaker, and the LOD of AgNCs mixed with R6G reaches 10^10^ M in Figure 5d. This can be attributed to the existence of plenty of sharp corners and edges, as confirmed from the TEM images and absorption spectra.

### 3.2. Detection of Azo Dye-Derived from Tyr by SERS

**Figure 6:**
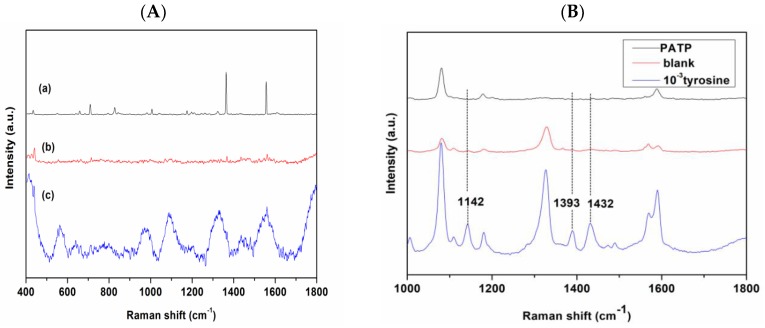
(**A**) Raman spectra of (**a**) Tyr solid; (**b**) Tyr (10^−3^ M) in AgNCs; and (**c**) AgNCs substrate; (**B**) SERS spectra of azo dye-derived from Tyr at 10^−3^ M, 10^−3^ M *p*-amino thiophenol (PATP) aqueous solution, and control blank sample without Tyr.

The normal Raman scattering (NRS) of Tyr is weak and the affinity of Tyr (10^−3^ M) to AgNCs is also rather weak. The NRS and SERS signals of Tyr, respectively, and the Raman spectrum of AgNCs substrate are shown in Figure 6A. It can be seen that Tyr without any modification is difficult to detect with SERS. To solve the problem of Tyr detection, we proposed a method for the indirect detection of Tyr based on azo coupling. PATP is one of the most important probe molecules in SERS research, as it can produce unique and strong SERS signals; however, it is also prone to many abnormal SERS signals [33]. In order to avoid abnormal SERS signal of the PATP molecule, Tyr was not added under the same experimental conditions as that of the control experiment (Figure 6B). Note that we could not find peaks at 1432, 1393, and 1142 cm^−1^ in the spectrum. Therefore, it can be confirmed that controlling the temperature and reaction conditions can prevent the self-coupling reaction of PATP, thus eliminating the possibility that the SERS signal is derived from PATP self-coupling. In case of Tyr (10^−3^ M) based on azo coupling, three peaks appeared at 1432, 1393, and 1142 cm^−1^, with the peak at 1432 cm^−1^ showing a strong intensity, owing to ‒N=N‒ stretching vibration of the trans isomer [34], indicating the existence of azo dye. The strong band at 1393 cm^−1^ was assigned to the ‒C‒C‒ within the phenyl rings coupling to the N=N stretch, and the peak at 1142 cm^−1^ corresponds to C‒N stretching vibration. Thus, the SERS spectra provide abundant fingerprint information for Tyr-derived azo products.

### 3.3. Detection of Azo Dye Derived from Tyr by D-SERS

**Figure 7:**
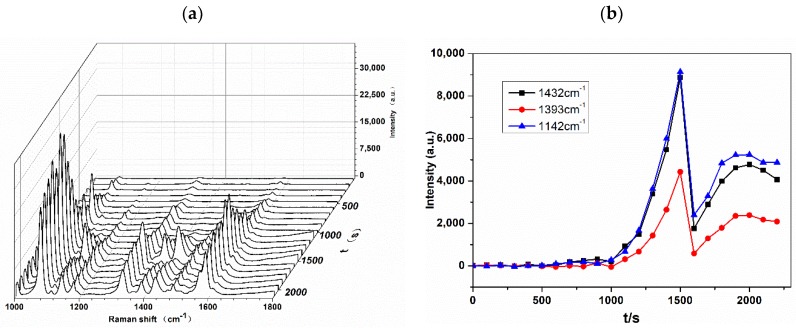
(**a**) Map of dynamic SERS (D-SERS) spectra of azo dye derived from Tyr (10^−6^ M) from 0 s to 2100 s across an AgNCs substrate; (**b**) SERS intensity at specific Raman modes of 1432, 1393, and 1142 cm^−1^ for azo dye derived from Tyr (10^−6^ M) obtained from D-SERS measurement.

Further, the optimal time for SERS measurement should be determined. A super-hydrophobic substrate has excellent analyte-enriching capability for SERS detection, and it can also be used for D-SERS. Figure 7a shows the three-dimensional spectrum of SERS intensity as a function of time, exhibiting the dynamic process of transforming the analyte from the wet state to the dry state. Figure 7b shows the variation in SERS absolute intensity for the specific vibration modes of 1432, 1393, and 1142 cm^−1^ from the D-SERS measurements from 0 s to 2100 s. The continuous time acquisition process has the advantage of real-time capturing and monitoring of the changes in SERS signals. It is observed that SERS is unable to detect beyond 1000 s, since no effective hot spots were formed in the initial sol phase. From the statistical data points, it is shown that we do not have to complete the test in 2 min, as reported previously. Although the SERS signal intensity is the strongest at about 1500 s, the time is too short to be measured accurately. With solvent evaporation, the spectral intensities were stabilized after 30 min, and the analyte concentrated into a small spot, which is in the dry state. Therefore, all of our experiments were performed in the dry state to reduce errors. The Tyr concentration was calculated using the intensities of the three characteristic peaks.

### 3.4. SERS Sensitivity of Azo Dye Derived from Tyr

**Figure 8:**
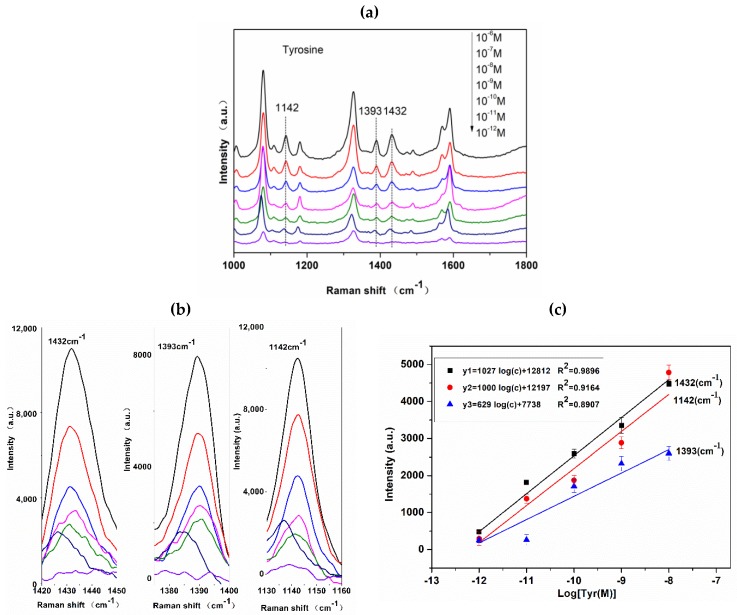
(**a**) SERS spectra of azo dye with various concentrations of Tyr; (**b**) Highlighted SERS spectra range of Tyr-derived azo dye with various concentrations at 1432, 1393, and 1142 cm^−1^; and, (**c**) Correlation between the intensities of three typical peaks of Tyr-derived azo dye at 1432, 1393, and 1142 cm^−1^ and Tyr concentration.

It has been reported that the high E(electric)-field localized at the corners of AgNCs and the chemisorption of analyte onto the sharp corners and edges of AgNCs may induce changes in the molecule’s polarizability, thus contributing to the total spectral enhancement of the analyte [17]. In addition, the micro-sodium structure with hierarchical pores guarantee a sufficient hydrophobicity of the silver film. Analytes with femtomolar concentrations in an aqueous droplet could be detected after being enriched on the super-hydrophobic silver film [35]. Therefore, combining the advantages of AgNCs with a super-hydrophobic substrate to detect azotized Tyr can greatly increase the LOD. Under the optimized experimental conditions and parameters, the sensitivity of Tyr based on azo coupling reaction were evaluated. The spectra of azo dye with different concentrations of Tyr are shown in Figure 8a. With a decrease in Tyr concentration, the intensities of the three typical peaks at 1432, 1393, and 1142 cm^−1^ gradually weakened. It shows that the detection limit of this method for azotized Tyr can reach the picomole levels; we regard it as a practical LOD for Tyr. The changes in the intensities of the typical peaks that are attributed to the Tyr-derived azo dye at 1432, 1393, and 1142 cm^−1^ with different Tyr concentrations are shown in Figure 8b. When the concentration ranges from 10^−6^ to 10^−7^ M, the peak intensities are particularly strong. In this range, most hot spots are occupied by azotized Tyr. When the concentration ranges from 10^−7^ to 10^−12^ M, which is termed the quantification region, the intensity is positively correlated to the log concentration of Tyr, and the results show good linearity in the ranges of 10^−7^–10^−12^ M (Figure 8c).

### 3.5. Application for Urine Detection

**Figure 9:**
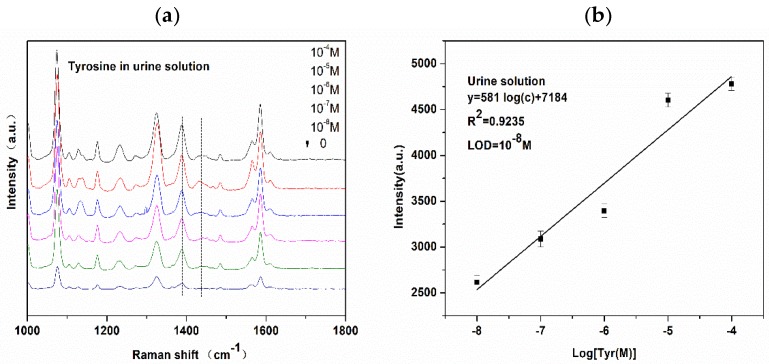
(**a**) SERS spectra of azo dye against various concentrations of Tyr in urine solution of normal human; (**b**) Intensity of the band at 1389 cm^−1^ versus log concentration of Tyr in urine solution.

To further investigate the feasibility of this proposed method for Tyr detection, normal human urine was chosen as a model for biological applications. In recent years, the Tyr urine test project has been included in the clinical testing project. The main components of urine are water, urea, uric acid, and inorganic salts. In this complex system, Tyr (10^−8^ M) in urine is detected without separation. Firstly, the morning urine was taken from healthy volunteers, centrifuged, and diluted with different concentrations of Tyr from 10^−4^ M to 10^−8^ M. The derivatization product, which was formed by the diazo coupling reaction, was mixed with the AgNCs substrate and detected on a super-hydrophobic silver film. The SERS spectra of different concentrations of Tyr-derived azo dye in urine solution and the blank sample as a control are shown in Figure 9a. Note that the three typical peaks of azo bonds are rather weak and slightly shifted. According to literature, the peak at 1133 cm^−1^ originates from C‒N stretching of uric acid [36], and that at 1142 cm^−1^ results from C‒N stretching vibration of Tyr-derived azo dye. However, in the urine solution, the C‒N peak shifted from 1142 cm^−1^ to 1128 cm^−1^, possibly owing to uric acid in the urine. Additionally, the other two characteristic peaks have also shifted, and the band at 1389 cm^−1^ arising from ‒C‒C‒ within the phenyl rings coupling with N=N stretch was selected for quantitative analysis. The peaks at 1440 cm^−1^ appeared due to ‒N=N‒ stretching vibration of the trans-isomer; the peak broadened, and the SERS signal intensity was weaker than that of the same concentration of Tyr in aqueous solution. Thus, we can infer that this phenomenon is caused by urea in urine. Urea can react with sodium nitrite [37]; therefore, during the diazo-coupling reaction, PATP reacts with sodium nitrite under acidic conditions to produce diazo compounds. If the reaction is incomplete, urea absorbs some sodium nitrite. In this complex system, PATP and urea play a competitive role. In the blank control sample (urine of normal people), traces of Tyr were detected. It is reported that the content of Tyr in normal human urine is 167 μmol/24 h, and the Tyr content differs for normal people with different ages. The urine sample that we selected in the experiment belongs to normal young people. The lowest detectable concentration of Tyr in the urine solution was 10^−8^ M; this LOD is sufficient for the detection of Tyr in the urine of normal people. As shown in Figure 9b, good linearity was observed when the intensity at the wavelength of 1389 cm^−1^ was plotted against the log concentration of Tyr from 10^−4^ M to 10^−8^ M in urine. In order to verify the accuracy of the results, the recovery test was conducted by preparing samples in different concentration in the lineal range, obtaining the corresponding SERS signal, and then calculating the ratio of measured concentration and true concentration, which is recovery (%) shown in Table 1.

## 4. Conclusions

In summary, we have demonstrated a SERS-based indirect method for Tyr detection by using AgNCs and a super-hydrophobic silver film. The method provided high spectral sensitivity for the detection of low concentration of Tyr, with the detection limit down to the pymole levels in solution. Before the experiments, a simple and sensitive SERS substrate that was based on AgNCs was used for the detection of R6G, and SERS enhancement using R6G as a model molecule was achieved at 10^−10^ M. Moreover, the target substance could be highly concentrated using a super-hydrophobic silver film to achieve the effective detection of low-level target molecules while greatly improving the sensitivity. Then, we determined the optimal time for analyte detection by D-SERS, which revealed that analyte detection using AgNCs SERS measurement is the best in the dry state. The proposed approach is simple, involves low sample consumption, and it does not require any separation process. Additionally, the results that were obtained from the analysis of urine of normal people confirmed the feasibility of our method in complex systems.

## Figures and Tables

**Table 1:** Recovery test of Tyr in the urine.

Samples	True concentration (mol/L)	Recovery (%)
Tyr in the urine	2.50 × 10^−6^	135.2
	2.50 × 10^−7^	110.0
